# Plasma protein increase as a chronological aging factor in healthy toy poodles

**DOI:** 10.1038/s41598-025-26154-2

**Published:** 2025-11-26

**Authors:** Satoru Ozaki, Yoshiko Honme, Seiichiro Higashi, Kouya Hattori, Masashi Morifuji, Eriko Mizuno, Minoru Yoshida, Takashi K. Ito

**Affiliations:** 1 Wellness Science Labs, Meiji Holdings Co., Ltd., Hachioji, Tokyo, Japan; 2 TSUKUBA PET Professional School, Tsukuba, Ibaraki Japan; 3 TSUKUBA Wanwan Land, Tsukuba, Ibaraki Japan; 4 Drug Discovery Seeds Development Unit, RIKEN Center for Sustainable Resource Science, Wako, Saitama Japan; 5 Faculty of Food and Nutritional Science, Toyo University, Asaka, Saitama Japan; 6 Office of University Professors, The University of Tokyo, Tokyo, Japan

**Keywords:** Plasma protein, Aging, Dogs, NAD⁺, Gut microbiota, Biochemistry, Microbiology, Zoology, Biomarkers

## Abstract

Although extensive research has examined aging markers in larger dog breeds, little is known about small breeds. This study assesses the relevance of aging biomarkers examined in larger breeds and other biological species, focusing on toy poodles (N = 40) as a model of small breeds and retrievers (N = 17) serving as a large-sized reference. Healthy individuals with no significant health declines for up to a year post-data collection were studied for age-related changes in various parameters, excluding disease factors. Our cross-sectional analysis identified significant correlations between age and increases in plasma protein concentration and amylase levels across both breeds, with breed-specific age-related declines in vaccine responses to various viruses observed only in toy poodles. Longitudinal analysis over one year confirmed a significant temporal increase in plasma protein in toy poodles, with a similar, albeit non-significant, trend in retrievers. Unlike in other species, nicotinamide adenine dinucleotide levels and fecal microbiota showed no age-related changes. Additionally, the previously reported frailty index correlated with age in retrievers but not in toy poodles. Notably, including deceased individuals during the study strengthened correlations. These results suggest plasma protein increase as a chronological aging factor in toy poodles and enhance our understanding of aging in healthy small dog breeds.

**Supplementary Information:**

The online version contains supplementary material available at 10.1038/s41598-025-26154-2.

## Introduction

Dogs have been domesticated for over 10,000 years and have long been integral members of human society^1^. Selective breeding has produced a variety of inbred lineages, resulting in approximately 400 pet dog breeds^2^. Due to strong artificial selection, domesticated dogs have become one of the most morphologically diverse mammalian species, with significant variation in size among breeds. The average adult dog weight varies more than 30-fold from breed to breed. Based on this size diversity, breeds may be classified as large (average adult weight > 25 kg), medium (10–25 kg), or small (< 10 kg)^3^. Size influences lifespan and aging rates in each breed, with an inverse correlation between size and lifespan^4,5^. Different breeds also exhibit varying rates of age-related diseases^6^. Canine breed size is positively associated with IGF-1 levels^7^, and slower aging progression in small breeds is attributed to relatively low IGF-1 levels^6,8^. Lowering IGF-1 signaling has been shown to increase lifespan in model organisms^9^. These findings highlight the need for studies across different breed sizes to better understand aging in pet dogs.

Multiple reports exist on factors associated with aging in dogs. It has been reported that DNA methylome analysis can estimate canine chronological age^10,11^. Several studies measuring blood biochemical indices have reported correlations between age and increased levels in total protein, globulin, ALT, albumin, and glucose^12–15^. While these studies have found common relationships between each factor and age, they are limited to large and medium breeds^12,13^ or to populations of multiple breeds in which these sizes are predominantly present^14,15^. While the population of small-sized breeds has increased in recent years, it remains largely unclear whether these findings can be adapted to small dogs or if they have different aging profiles. Additionally, many studies are cross-sectional, and there are limited reports analyzing aging-related factors in the same population through longitudinal analysis.

It has been reported that in humans, immune function declines with age and vaccine response decreases^16–19^. This decreased reactivity has also been observed for vaccines against COVID-19 across a wide geographic and racial spectrum^20,21^, suggesting decreased reactivity to new antigens. Post-vaccination antibody titers have also been reported to decrease in large and medium-sized dogs as they age, but reports on small breeds are limited^22,23^.

Frailty is a condition commonly seen in the elderly that increases the risk of falls, disability, hospitalization, and death^24^. In humans, several indices have been proposed to objectively assess the degree of frailty, and these are used clinically for health management^24–26^. A method for calculating the frailty index has also been proposed for dogs, showing a modest correlation between aging and the index, and a strong association with mortality regardless of age^27^. However, the number of studies on small dogs is relatively small, and the association is weak^27^.

NAD⁺ (nicotinamide adenine dinucleotide) is a coenzyme involved in energy metabolism, DNA repair, and cell signaling^28–30^. It has been reported that NAD⁺ levels in vivo decrease with aging common in eukaryotes including humans^31–35^, rodents^35,36^, and nematodes^36^. In mice, the intake of NAD⁺ precursors increases NAD⁺ levels and improves tissue functions and metabolism^36–38^. However, no reports on the relationship between aging and in vivo NAD⁺ levels in dogs have been published at the time of this manuscript’s submission.

The composition of the human gut microbiota has been associated with various poor health conditions and diseases such as obesity, diabetes, and inflammatory diseases^39,40^. The human gut microbiome also changes with age, with a decrease in *Bifidobacterium* and *Faecalibacterium* and an increase in Enterobacteriaceae reported with aging^41,42^. Additionally, frail humans show a decrease in *Faecalibacterium* and an increase in Enterobacteriaceae compared to non-frail individuals, exhibiting a trend similar to chronological aging^43,44^. These findings suggest that the human gut microbiota is linked to health and aging. In contrast, fewer studies have investigated the age-related changes in the canine gut microbiota, and consistent patterns have yet to be established^45–49^.

Here, we aim to assess changes associated with chronological aging in healthy dogs by measuring multifaceted parameters. Toy poodles were analyzed as representatives of small dogs, and retrievers were analyzed as representatives of large dogs for comparison. To enhance the study’s sensitivity despite the small sample size, we employed a unique cohort of dogs raised under uniform housing conditions with a consistent diet throughout their lives. This controlled environment minimizes common confounding factors such as diet, environment, and social stress, which often affect aging biomarkers in dogs. Using both cross-sectional and longitudinal approaches, we measured blood biochemical indices, blood NAD⁺ levels, fecal microbiome, antibody titers as vaccine responses against multiple viruses, echocardiographic measurements, and the frailty index as candidate parameters. All parameters, except for antibody titers, were measured twice with a six-month interval between measurements. We analyzed the relationship between age and various indicators. Indicators with the strongest correlations were subjected to longitudinal analysis over a one-year period to evaluate whether there were consistent changes with age over time.

## Materials and methods

### Animals

Toy poodles, golden retrievers, and Labrador retrievers kept as companion animals at the animal-interaction section of the zoological park in Ibaraki Prefecture, Japan, were recruited for the study with the consent of the owners. The cohort comprised dogs born in-house and others from external facilities. No dogs had been involved in other research. During the day, the dogs were allowed to move and play with humans under staff supervision inside the zoo, whereas at night each dog was housed individually in a kennel cage with bedding. All dogs were fed the same commercial dry food (Dog Meal, Cainz, Japan; marketed as a complete and balanced maintenance diet). The guaranteed analysis is crude protein ≥ 18.0%, crude fat ≥ 8.0%, crude fibre ≤ 6.0%, crude ash ≤ 10.0%, moisture ≤ 10.0%. We excluded dogs with pre-existing diseases, those diagnosed by a veterinarian with poor health status at the start of the study, and those that died within 24 months from the start due to the possibility of being unhealthy. Animals that became pregnant during the study were excluded upon confirmation of pregnancy. In some analyses, participants were stratified based on their age at the start of the study. Toy poodles under 7 years old and retrievers under 6 years old were classified as "Adult," while participants older than these thresholds were classified as “Senior”^23,50^. The two breeds of retriever were merged in the data analysis.

### Blood sampling

The participants were fasted from 18:00 on the day before blood collection until sampling, which was performed between 10:00 and 14:00 the next day; water was provided ad libitum. Accordingly, the fasting duration ranged from approximately 16 to 20 h across all animals^51,52^. Blood samples were collected using a syringe from the cephalic vein. After collection, the blood samples were transferred to silicon-coated blood collection tubes (#365963, Becton, Dickinson and Company, NJ, USA) for biochemical and antibody titer testing, and to evacuated tubes containing sodium fluoride, citric acid, sodium citrate, and EDTA-2Na (VP-FH051K, Terumo, Japan) for NAD^+^ quantification.

### Plasma biochemical analysis

Plasma was obtained by centrifuging the tubes at room temperature at 3000 rpm for 10 min, after which the supernatant was collected as plasma. Plasma was kept refrigerated below 10 °C for 24 to 32 h until biochemical analysis and antibody titer measurement. The concentrations of total protein (TP), albumin (ALB), lipase (LIP), calcium (Ca), and total bilirubin (TBIL) were measured by the biuret method^53^, bromocresol green method^54^, DGGR substrate chromogenic method^55^, o-cresolphthalein complexone method^56^, and chemical oxidation method^57^, respectively. Globulin (GLO) concentration was calculated by subtracting the ALB concentration from the TP concentration. The albumin/globulin ratio (ALB/GLO) was then calculated by dividing the ALB concentration by the GLO concentration. Blood urea nitrogen (BUN)^58^, creatinine (CRE)^53^, blood glucose (GLU)^59^, total cholesterol (TC)^60^, triglycerides (TG)^61^, total bile acids (TBA)^62^, and inorganic phosphorus (P)^63^ were measured by enzymatic methods. Aspartate aminotransferase (AST)^64^, alanine aminotransferase (ALT)^65^, and amylase (AMY)^66^ were measured by the Japanese Society of Clinical Chemistry standardization method. Sodium (Na), potassium (K), and chloride (Cl) were measured by the electrode method^53^.

### Quantification of whole blood NAD^+^ concentration

The processing method for blood samples for NAD^+^ quantification followed the protocol described in a previous report^67^ with the following modification: 20 µL of whole blood were mixed immediately with 980 µL ice-cold 55% MeOH and stored at − 80 °C. After thawing, a 13C5-NAD^+^ internal standard was added, the extract was ultrafiltered (10 kDa cut-off), half of the filtrate was dried, reconstituted in 1 mL 10 mM ammonium acetate (instead of water), diluted 1:10, and analyzed by LC–MS/MS. LC–MS/MS analyses were performed using a Shimadzu HPLC system (Shimadzu, Japan) coupled with a QTRAP4500 mass spectrometer (AB Sciex, MA, USA). Chromatographic separation was achieved on a Hypercarb column (2.1 mm × 100 mm, 3 μm particle size, Thermo Scientific, WI, USA). The column temperature was maintained at 60 °C. The mobile phase consisted of 10 mM ammonium acetate with 0.05% (v/v) ammonium hydroxide in water (A) and 0.05% (v/v) ammonium hydroxide in acetonitrile (B), delivered at a flow rate of 0.2 mL/min. The injection volume was 3 μL. The gradient elution started with 5% B for the first 1.8 min, increased to 54% B from 1.8 to 14 min, then to 90% B from 14.0 to 14.10 min, held at 90% B until 17.1 min, decreased linearly to 5% B from 17.1 to 17.2 min, and remained at 5% B until 32.2 min. The MS/MS analysis was performed in positive ESI mode. The detection of NAD^+^ was performed by multiple reaction monitoring. The ions were selected in the first quadrupole (Q1) and collided with nitrogen gas in the second quadrupole (Q2), and the product ions were detected in the third quadrupole (Q3). The m/z value of the precursor ions detected at Q1 was 664.2 (NAD^+^) and 669.1 (stable isotope-labeled NAD^+^). The collision energy at Q2 was 38 V. The m/z values for the product ions detected at Q3 were both 428.2 (NAD^+^ and stable isotope-labeled NAD^+^).

### Echocardiography

Echocardiography was performed using a LOGIQ P6 (GE Healthcare, USA). IVSd (mm), LVIDd (mm), LVIDs (mm), and LVPWd (mm) were measured in M-mode using the right parasternal left ventricular short-axis cross-sectional image in the right lateral recumbent position with hand-holding without instruments. EDV (ml), ESV (ml), EF (%), SV (ml), and FS (%) were calculated from these measurements. Additionally, the E/A ratio was measured using the pulsed Doppler method for inflow blood flow at the mitral valve, using a left parasternal apical quadrant cross-sectional image in the left lateral recumbent position. Each measurement was performed 2–5 times, and the average value was recorded.

Abbreviations of each parameter are as follows:

IVSd: Interventricular Septal thickness in diastole

LVIDd: Left Ventricular Internal Dimension in diastole

LVIDs: Left Ventricular Internal Dimension in systole

LVPWd: Left Ventricular Posterior Wall thickness in diastole

EDV: End-Diastolic Volume

ESV: End-Systolic Volume

EF: Ejection Fraction

SV: Stroke Volume

FS: Fractional Shortening

E/A ratio: Early diastolic mitral inflow velocity/Atrial systolic mitral inflow velocity ratio

### Vaccination and measurement of antibody titer

At 6 months after the start of the study (6 m), the participants received a vaccination with Vanguard Plus 5CV/L (Zoetis, NJ, USA). This formulation included vaccines for distemper, parvo, and adeno viruses with potencies of over 10^3.5^, 10^6.4^, and 10^5.5^ TCID50, respectively. All included dogs had received the same vaccine, exactly 12 months before the vaccination of this study; two toy poodles that had been vaccinated on a different schedule were excluded from the analysis because of other exclusion criteria. Blood samples were collected before vaccination (6 m), 1 month after vaccination (7 m), and 6 months after vaccination (12 m) to measure antibody titers for distemper, parvo, and adeno viruses. Plasma was obtained using the same method described in the biochemical test section. Antibody titers were measured by fluorescence immunoassay with Vcheck Ab (Bionote, Korea)^68^.

### Frailty index

The frailty index was evaluated using the method described in a previous report^27^. Briefly, 33 items related to frailty were assessed by veterinary nurses and a veterinary physician. Some health deficits were assigned a score of 0 (absent) or 1 (present), while other deficits were evaluated using a semi-quantitative scale as absent (score = 0), mild (score = 0.5), or severe (score = 1). Prior to data collection, the veterinary nurses and physician jointly calibrated the severity levels and scoring criteria for each frailty item, ensuring consistent ratings within and between assessors. The frailty index was calculated by dividing the sum of the scores by the total number of items (33).

### Fecal sampling and DNA extraction

Feces naturally excreted were collected into a feces container (Sarstedt) within 60 min of defecation, frozen at -18 °C or below, stored at that temperature for up to 96 h, and subsequently transferred to -80 °C until DNA extraction. Whole genomic DNA from feces was extracted according to “protocol Q” from a previous report^69^, with slight modification. First, 20 mg of feces (instead of 150–200 mg) were processed in 1 mL Buffer ASL containing 0.3 g of 0.1 mm zirconia beads. Second, a two-step extraction was introduced: after the initial bead-beating, heating (95 °C, 15 min), and centrifugation, the pellet was resuspended in 300 µL Buffer ASL, subjected to the same heat and bead-beating cycle, re-centrifuged, and the second supernatant was pooled with the first before ammonium-acetate precipitation and column purification.

### 16S rRNA gene sequencing library preparation and sequencing

The V3-V4 region of the bacterial 16S rRNA gene was amplified using genomic DNA as a template and primers 5′-TCGTCGGCAGCGTCAGATGTGTATAAGAGACAGCCTACGGGNGGCWGCAG-3′ and 5′-GTCTCGTGGGCTCGGAGATGTGTATAAGAGACAGGACTACHVGGGTATCTAATCC-3′^70^. Template genomic DNA concentration was measured using Quant-iT™ dsDNA Assay Kits (Thermo Fisher Scientific) and adjusted to 5 ng/μL. 16S Metagenomic Sequencing Library Preparation was performed according to the manufacturer’s instructions (Illumina, CA, USA). The DNA library was sequenced using a MiSeq System (Illumina) with a 2 × 300-base-pair protocol.

### Analysis of 16S rDNA sequencing data

Sequencing data were analyzed using Qiime2 (version 2020.11)^71^. To trim the primer region from raw sequences, the Cutadapt plugin in Qiime2 was used^72^. Sequences without the primer region were processed for quality control, paired-end read joining, chimera filtering, and amplicon sequence variant (ASV) table construction using the DADA2 algorithm^73^. For each representative sequence of ASV, BLAST^74^ was used to assign the taxonomy based on the SILVA database (version 138)^75^. After random sampling of 14,000 reads using the feature-table plugin^76^, conversion of compositional data and diversity analysis was performed.

### Statistical analysis

AMY, LIP, ALT, and AST were analyzed using log_10_-transformed values. R (ver. 4.3.2) was used for linear mixed model analysis of TP, ALB, GLO, ALB/GLO and AMY using the lme4 package. TP, ALB, GLO, ALB/GLO and AMY were modelled as a function of the time of measurement (0, 6, and 12 m), with time included in the model as a fixed effect and individual as a random effect. Changes of each indice per unit time per month were calculated and tested for significant differences in change over time. Permanova analysis with Weighted UniFrac Distance was used to test for significant differences in fecal microbiota β-diversity between groups. GraphPad Prism 8 was used for Mann–Whitney U-test and Spearman’s rank correlation analysis. Statistical significance was set at p < 0.05, and p values from 0.05 to 0.10 were regarded as marginally significant.

## Results

### Study subjects

**Fig. 1:**
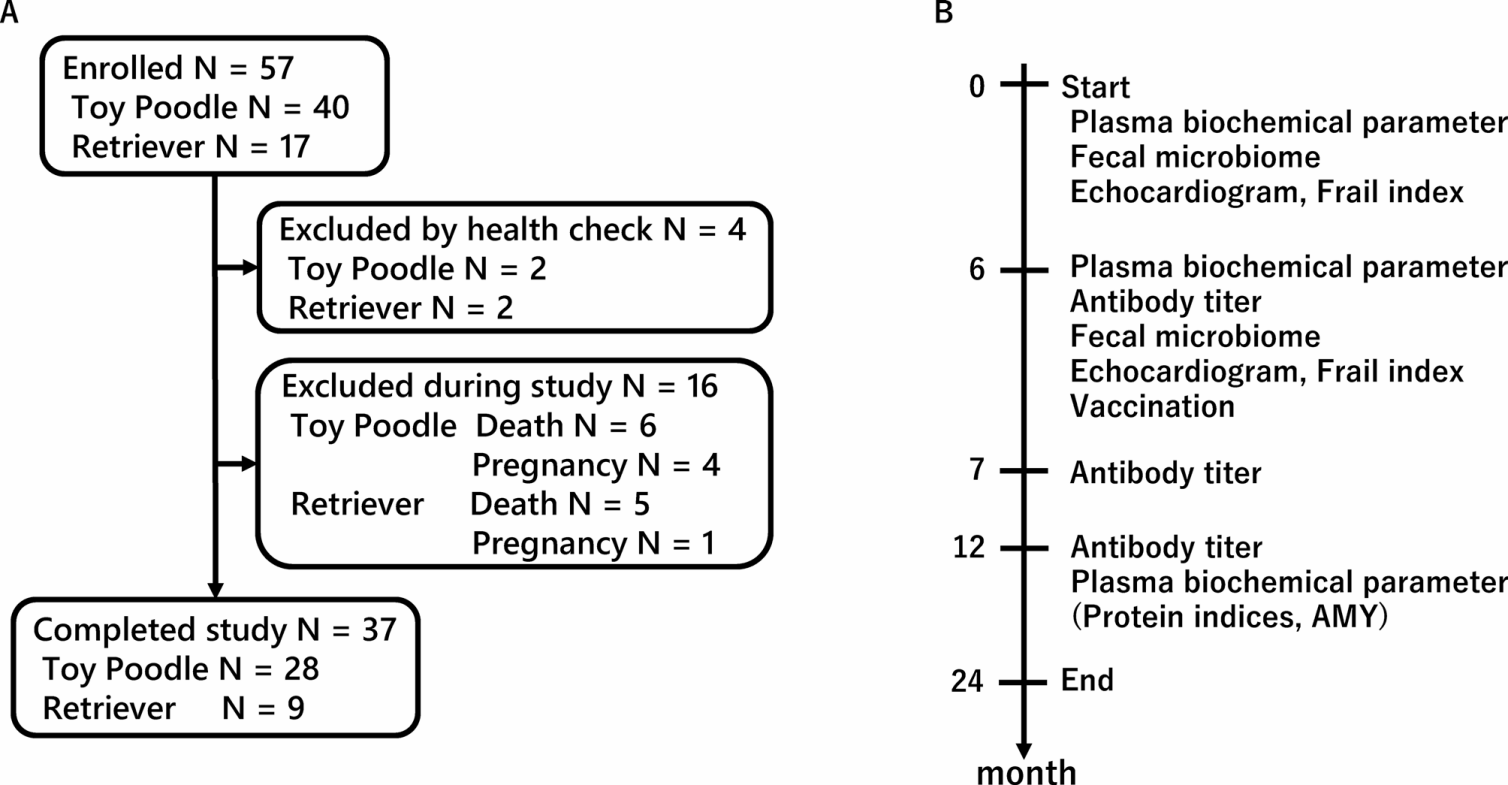
Individuals and schedule. (**A**) Number of individuals enrolled, excluded, and completed in the trial. N: Number of individuals. (**B**) Schedule of the trial.

Forty toy poodles, eight golden retrievers, and nine Labrador retrievers were recruited for this study (Fig. 1A). Two toy poodles and two retrievers were excluded due to health check results at the start of the study. During the study, six toy poodles and five retrievers were excluded due to death, and four toy poodles and one retriever were excluded due to pregnancy. Twenty-eight toy poodles (nine males, 19 females) and nine retrievers (four golden, five Labrador; three males, six females) successfully completed the study. Among the dogs who completed the study, six toy poodles, three golden retrievers, and two Labrador retrievers were biologically related as siblings or as parent–offspring pairs. In addition, five toy poodles and one Labrador retriever had been spayed. The age of the participants who completed the study ranged from 1 to 14 years at the start. The median ages for toy poodles and retrievers was 6.6 years (range 1.3–14.2 years) and 5.6 years (range 2.8–9.7 years), respectively. Based on the threshold, 15 toy poodles and five retrievers were assigned as adults, while 13 toy poodles and four retrievers were assigned as seniors. The median body weight of the completed toy poodles and retrievers at the start were 4.8 kg (range 2.8–8.7 kg; SD 1.2) and 24.0 kg (range 18.3–34.2 kg; SD 4.5), respectively.

### Plasma biochemistry tests

Blood was collected from each animal at the start of the study (0 m) and six months later (6 m), and 20 plasma biochemical indices were measured (Fig. 1B). Fifteen of the 20 indices were compared to the reference range^77^, and the mean values of these 15 indices measured in this study were all within the reference range. These results indicate that the blood biochemical indices in the present study population do not deviate significantly from those in other healthy cohorts.

**Fig. 2:**
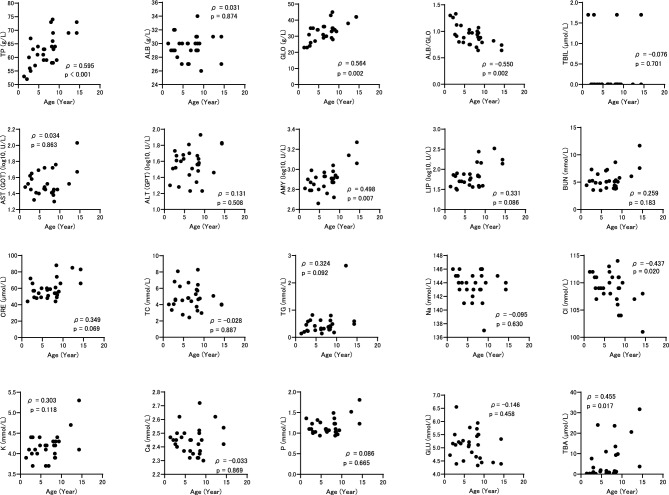
Relationship between age and blood biochemical indicators in toy poodles at 0 m. ρ represents the Spearman rank correlation coefficient between age and each indicator.

**Fig. 3:**
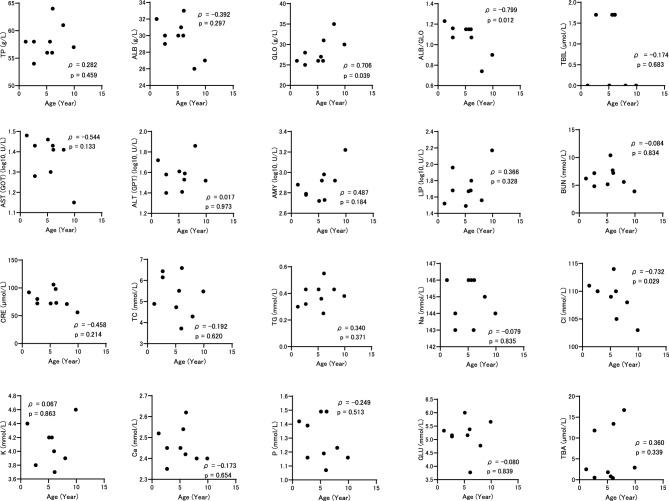
Relationship between age and blood biochemical indicators in retrievers at 0 m. ρ represents the Spearman rank correlation coefficient between age and each indicator.

Correlation analysis between each indicator and age was performed in both the toy poodle and retriever populations. In the toy poodles, TP, GLO, and AMY showed a significant (*p* < 0.05) positive correlation with age, whereas ALB/GLO showed a significant negative correlation with age at both 0 m and 6 m (Figs. 2, S1). TBA and Cl showed significant positive and negative correlations with age at 0 m only, respectively. In retrievers, GLO showed a significant positive correlation with age at 0 m, and a positive correlation at 6 m but not significant (Figs. 3, S2). Cl and ALB/GLO showed a significant negative correlation with age at 0 m only. TP and AMY, which correlated with age in toy poodles, showed positive correlation coefficients in retrievers, although they were not significant at 0 m and 6 m. These data suggest that blood TP, GLO, ALB/GLO and AMY levels are linked to age, commonly observed regardless of the timing analyzed.

### Whole blood NAD⁺ concentration

To investigate the relationship between in vivo NAD⁺ concentration and age in dogs, we quantified the concentration of NAD⁺ in whole blood at 0 m and 6 m, and analyzed the correlation with age (Fig. S3). The mean concentrations of NAD⁺ in whole blood at 0 m and 6 m were 23.5 μM (range 15.0–40.5 μM) and 23.6 μM (range 16.2–36.7 μM) for toy poodles and 20.6 μM (range 15.6–26.8 μM) and 23.3 μM (range 18.3–26.5 μM) for retrievers, respectively. At 0 m, no significant correlation between age and NAD⁺ was identified in either toy poodles or retrievers (Fig. S3A,B). There were no significant differences in NAD⁺ concentrations among breeds at 0 m (Fig. S3C). The same trends were observed at 6 m (Fig. S3D–F). These results indicate that the concentration of NAD⁺ in canine whole blood is not significantly correlated with age and that there are no significant differences among toy poodles and retrievers.

### Frailty index

**Fig. 4:**
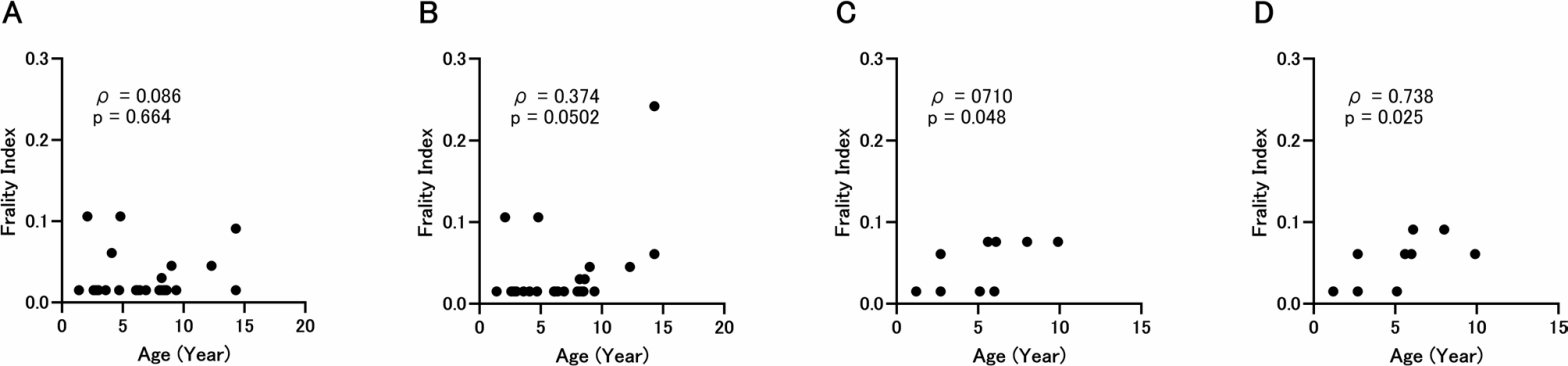
Relationship between age and the frailty index. (**A**) Correlation between age and the frailty index in toy poodles at 0 m. (**B**) Correlation between age and frailty index in toy poodles at 6 m. (**C**) Correlation between age and the frailty index in retrievers at 0 m. (**D**) Correlation between age and the frailty index in retrievers at 6 m. ρ represents the Spearman rank correlation coefficient between age and each indicator.

The same frailty index as previously reported^27^ was measured in our cohort and analyzed for correlation with age (Fig. 4). We found no significant correlation between frailty index and age at 0 m in toy poodles (ρ = 0.066, *p* = 0.66, Fig. 4A) and a positive correlation trend at 6 m (ρ = 0.374, *p* = 0.0502) (Fig. 4B). In retrievers, frailty index and age showed a significant positive correlation at both 0 m (ρ = 0.710, *p* = 0.048) and 6 m (ρ = 0.738, *p* = 0.025) (Fig. 4C,D). Since frailty index is positively related to mortality^27^, we analyzed the relationship between frailty index and age at 0 m, including individuals excluded due to death, and found that frailty index was positively correlated with age in toy poodles (Fig. S4A, ρ = 0.379, *p* = 0.030). In retrievers, the correlation coefficient with frailty index increased with the addition of deceased individuals (Fig. S4B, ρ = 0.865, *p* < 0.001). These results suggest that frailty index is positively correlated with age in retrievers in the present study population, and that in toy poodles it is weakly related to age in healthy individuals but positively related to mortality.

### Echocardiography

To investigate the relationship between cardiac functions and age, echocardiography was performed at 0 m and 6 m on toy poodles over 8 years old, and correlation analysis between each index and age was conducted (Figs. S5, S6). No significant correlation with age was observed for any of the 10 indices measured. However, EDV showed a trend of positive correlations with age at both 0 m and 6 m, and LVIDd and SV at 6 m only (*p* < 0.1).

### Vaccine antibody titer

**Fig. 5:**
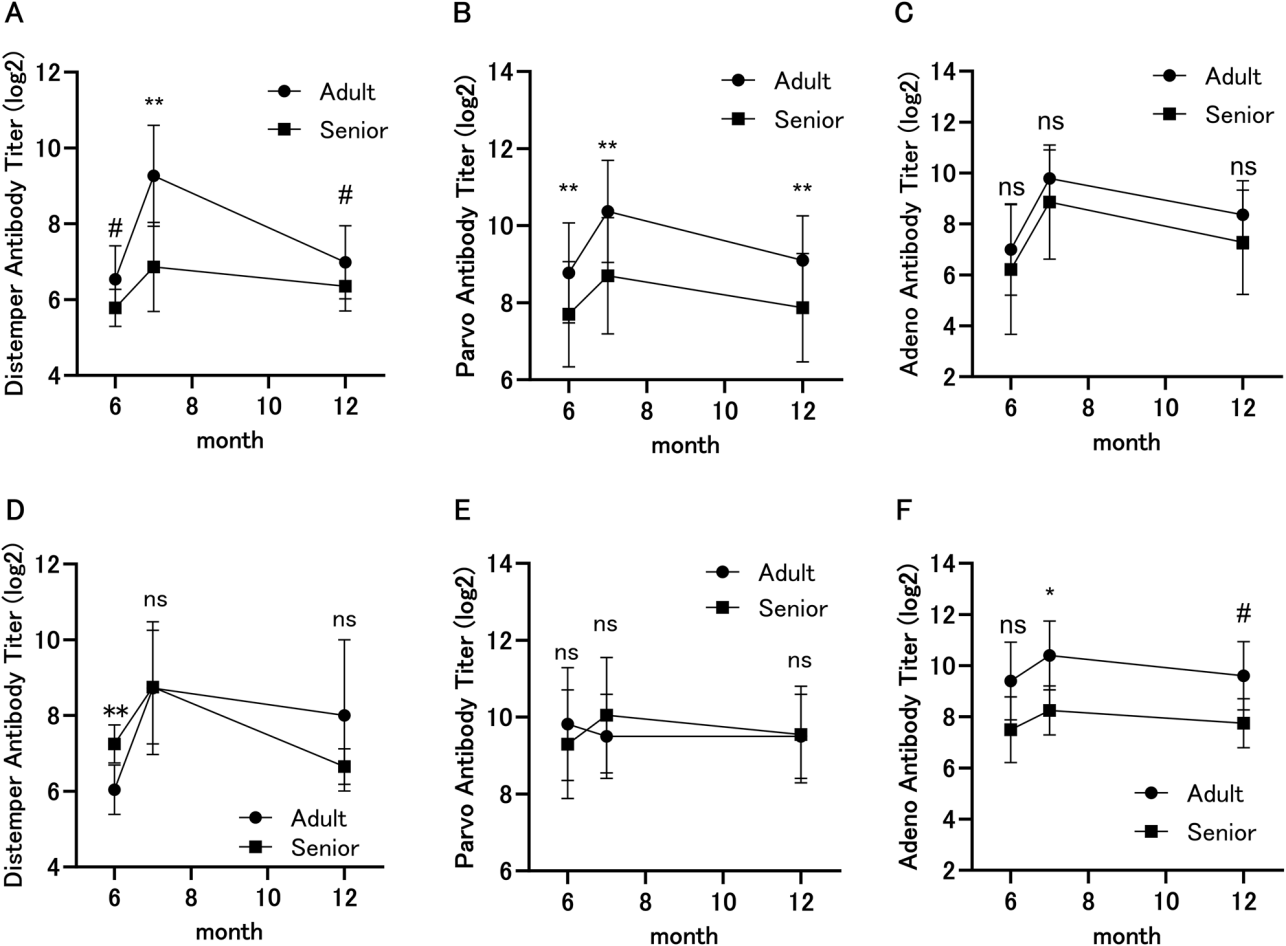
Effects of vaccination on distemper, parvo and adeno virus antibody titers. (**A**) Distemper virus antibody titer of toy poodles. (**B**) Parvo virus antibody titer of toy poodles. (**C**) Adeno virus antibody titer of toy poodles. (**D**) Distemper virus antibody titer of retrievers. (**E**) Parvo virus antibody titer of retrievers. (**F**) Adeno virus antibody titer of retrievers. Y-axis indicates logarithmically transformed antibody titer values. X-axis indicates the number of months since the start of the study. Vaccination was administered immediately after blood collection at 6 months. The Mann–Whitney test was used to test significant differences between the Adult and Senior groups at each time point. **p < 0.01, *p < 0.05, #*p* < 0.1, ns: *p* > 0.1.

To examine the relationship between aging and vaccine reactivity, antibody titers were measured before (6 m), one month after (7 m), and six months after (12 m) vaccination. Titers at 6 m reflect the persistence of the vaccine shot from a year ago, titers at 7 m reflect an acute vaccine reaction to a new shot, and titers at 12 m reflect the persistence of that effect. The measurements of the Adult (younger individuals) and Senior (older individuals) groups were compared. In toy poodles, distemper virus vaccine antibody titers were significantly lower in the Senior group at 7 m (*p* = 0.0011) and tended to be lower at 6 m (*p* = 0.059) and 12 m (*p* = 0.052) compared to the Adult group (Fig. 5A). Parvo virus vaccine antibody titers in toy poodles were significantly lower (6 m; *p* = 0.0047, 7 m; 0.0028, 12 m; 0.0079) in the Senior group than in the Adult group at all three time points (Fig. 5B). Adeno virus vaccine antibody titers were lower in Seniors than in Adults at all three time points, although none were significant (Fig. 5C).

In retrievers, in contrast to toy poodles, distemper virus vaccine antibody titers were significantly higher in the Senior group at 6 m (*p* = 0.0079), and there were no significant differences between the two groups at 7 m and 12 m (Fig. 5D). Parvo virus vaccine antibody titers in retrievers were not significantly different at 6 m, 7 m, and 12 m (Fig. 5E). Adeno virus vaccine antibody titers in retrievers were lower on average in Seniors than in Adults at all three time points and were significant at 7 m (Fig. 5F, *p* = 0.040).

### Fecal microbiome

**Fig. 6:**
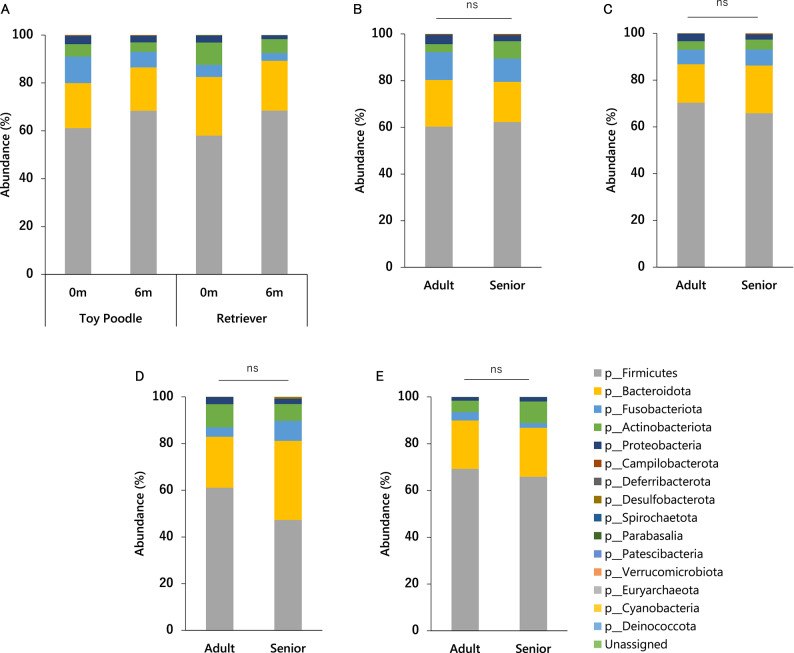
Average phylum-level composition of bacteria in dog feces. (**A**) Average composition according to breeds at 0 m and 6 m. (**B**) Average composition of bacteria in feces according to age groups of toy poodles at 0 m. (**C**) Average composition of bacteria in feces by age groups of toy poodles at 6 m. (**D**) Average composition of bacteria in feces by age groups of retrievers at 0 m. (**E**) Average composition of bacteria in feces by age groups of retrievers at 6 m. The significance of the difference between the Adult and Senior groups in β diversities was tested using the PERMANOVA test with the Weighted UniFrac Distance. ns: *p* > 0.05.

To evaluate the effects of age on the intestinal microbiota of poodles and retrievers, fecal samples were collected at 0 m and 6 m for microbiota analysis. The predominant bacterial phyla identified were Firmicutes, Bacteroidota, Fusobacteriota, Actinobacteriota, and Proteobacteria (Fig. 6A).

To examine the effect of age, the β-diversity analysis was performed for the Adult and Senior groups at 0 m and 6 m, and found no significant differences between Adult and Senior groups in both breeds at either time point (Fig. 6B–E, *p* > 0.1). In humans, an age-associated increase in Enterobacteriaceae and a decrease in *Bifidobacterium* and *Faecalibacterium* have been reported^41,42^. We compared the occupancy of these taxa in the Adult and Senior groups (Figs. S7, S8). In toy poodles, Enterobacteriaceae was significantly lower in the Senior group than in the Adult group only at 0 m (*p* = 0.0015), contrary to the human reports. No significant differences were detected in *Bifidobacterium* and *Faecalibacterium* between the groups at 0 m and 6 m (*p* > 0.1). Thus, no significant age-related effects on the fecal microbiome were noted in this cohort.

### Longitudinal analysis of plasma protein indices and AMY

From these cross-sectional analyses at 0 and 6 months, plasma TP, GLO, ALB/GLO and AMY were found to be significantly correlated with chronological aging at both time points in toy poodles. In addition, parvovirus antibody titer was significantly higher in Adult group than in Senior group before and after vaccination. The practicability of antibody titer as an indicator of aging is limited because its value fluctuates depending on the timing of vaccination. Therefore, we examined the validity of TP, ALB, GLO, ALB/GLO and AMY as chronological aging factors by following their changes over time.

**Fig. 7:**
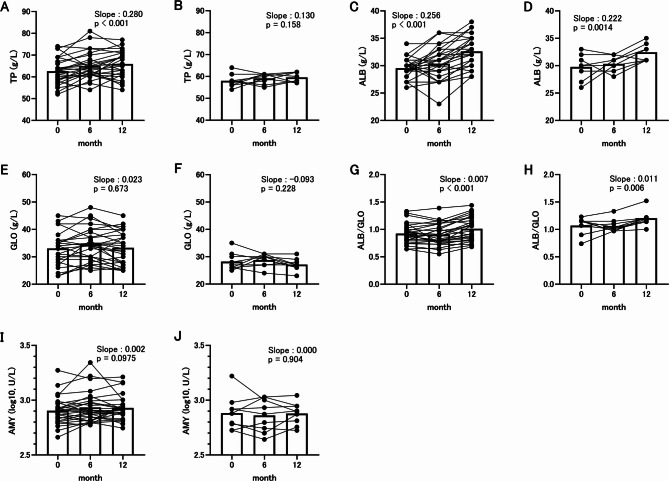
Longitudinal analysis of blood protein indices and amylase (AMY). (**A**) Total protein (TP) in toy poodles. (**B**) TP in retrievers. (**C**) Albumin (ALB) in toy poodles. (**D**) ALB in retrievers. (**E**) Globulin (GLO) in toy poodles. (**F**) GLO in retrievers. (**G**) Ratio of albumin per globulin (ALB/GLO) in toy poodles. (**H**) ALB/GLO in retrievers. (**I**) AMY in toy poodles. (**J**) AMY in retrievers. The values were measured at 0, 6, and 12 m. Dots represent measurements for each individual, and dots connected by lines indicate measurements for the same individual. White bars indicate mean values. Slope and p-value indicate the variability of the level of indices per month calculated by the mixed model and its significance, respectively.

For the population in this study, TP, ALB, GLO, ALB/GLO and AMY were also measured at 12 m from the start of the study, and changes per month were calculated (Fig. 7). The results showed that TP increased significantly over time in toy poodles (Fig. 7A, *p* < 0.001). In retrievers, TP increased over time, but not significantly (Fig. 7B, *p*  = 0.158). ALB increased significantly over time in both toy poodles (*p* < 0.001) and retrievers (p = 0.0014) (Fig. 7C, D). GLO showed no significant temporal change in both toy poodles and retrievers (p > 0.1, Fig. 7E, F). ALB/GLO increased significantly over time in both toy poodles (*p* < 0.001) and retrievers (p = 0.006) (Fig. 7G, H). In toy poodles, AMY increased over time, but not significantly (Fig. 7I, *p* = 0.098). In retrievers, AMY showed little variation over time (Fig. 7J, p = 0.904).

## Discussion

The primary objective of this study was to identify factors associated with chronological aging in small-sized dogs. We found that TP, GLO and AMY were positively correlated with age, and ALB/GLO showed the opposite trend in a cohort of healthy toy poodles. And TP continuously increased over a one-year period in this breed. Vaccine responses for several viruses declined in the older age groups. The frailty index was positively related to age only when the population included individuals who died during the study. No significant relationship with age was found for whole blood NAD⁺ concentration, echocardiographic indices, or fecal microbiome. Similar trends of an increase in TP, GLO and AMY were observed in retrievers. In contrast, vaccine antibody titers showed no consistent changes with age in retrievers.

Plasma biochemical indices are among the most frequently investigated in relation to aging in dogs^12–15^. Several indices, including TP, have been positively correlated with age in studies of mixed breeds or single medium or large breeds^12,14,15^. However, it remains unclear which of these indices are particularly linked to aging. Our findings that TP is correlated with aging in poodles in both cross-sectional and longitudinal analyses suggest that TP may be used as an aging indicator in small dogs, although further validation is required. A similar trend was also observed in retrievers, although the reference population was relatively small. Additional studies are needed to determine whether TP can also be applied to medium and large dogs. In laboratory animal models, mild suppression of protein synthesis has been strongly associated with increased longevity^78^. Future research should explore the causal relationship between elevated plasma protein levels and accelerated aging in pet dogs.

Regarding the relationship between blood protein composition and age in dogs, albumin (ALB), the most abundant protein in the blood, typically decreases in late aging or shows no strong correlation with age. In contrast, globulin (GLO), the second most abundant protein, generally increases with age^13–15^. In our cohort, which remained healthy at least 1 year after data collection, we found no significant correlation between age and ALB in either toy poodles or retrievers, while GLO was positively correlated with age in cross-sectional analysis. Conversely, ALB showed a significant increase longitudinally over the one-year period, whereas GLO exhibited little variation over time during the same period. This seemingly paradoxical pattern may be explained by the hypothesis that ALB gradually increases with age under healthy conditions, but its production is subsequently replaced by GLO expression as health status declines. Because the cross-sectional and one-year longitudinal findings for both ALB and GLO were not fully consistent, longer-term follow-up studies are warranted to clarify their age-related dynamics. Chronic kidney disease has been reported to cause hypoalbuminemia in dogs^79^. It is possible that ALB levels in this study focusing on healthy individuals were not affected because it did not include individuals with hypoalbuminemia.

In the present study, toy poodles showed a decrease in vaccine antibody titers with age, which is consistent with previous reports in other medium and large-sized breeds^22,23^. On the other hand, in retrievers, the vaccination antibody titers of the three viruses responded differently: Adeno virus antibody titers showed an age-related decrease, while Distemper virus and Parvo virus antibody titers showed no consistent age-related relationship. The cause of this is unknown, but it is possible that some individuals were subclinically infected, or vaccine reactivity might be unique to retrievers. Further studies on a larger scale including antibody titer measurements for other vaccine types are needed for a detailed analysis.

NAD⁺ levels decrease with age in worms, flies, and mice, and have also been reported to decrease in human tissues such as blood, brain, and skin in data from relatively small populations^31–33,80^. We were unable to find any prior reports quantifying in vivo NAD⁺ levels in dogs until the time of manuscript preparation. We measured whole blood NAD⁺ levels in the present study and found no significant relationship with age in either of the breeds. The mean NAD⁺ concentration in dogs was 23 μM (range 15–41 µM), which was close to the human NAD⁺ concentration of 26 μM (range not reported) measured by the same extraction method used in this study^67^. While these results do not rule out the possibility of a correlation between NAD⁺ levels and aging in other tissues, they do indicate that it is highly unlikely that NAD⁺ levels in whole blood can be used as a sensitive marker of aging in healthy dogs.

We evaluated the frailty index and echocardiography as indicators of health-related phenotypes that can be assessed by veterinarians and nurses. Regarding the frailty index, which has been reported to correlate with aging and mortality in a diverse population of large- and medium-sized breeds^27^, no significant relationship with age was found in the toy poodle population we evaluated. However, in an analysis including individuals who died during the study, the frailty index at study entry showed a significant positive correlation with age. In retrievers, the correlation coefficient was also higher in analyses that included deceased individuals. These results suggest that the frailty index might be a stronger predictor of mortality than chronological age.

Less is known about the relationship between the fecal microbiome and aging in dogs than in humans. In this study, the predominant bacterial phyla identified were Firmicutes, Bacteroidota, Fusobacteriota, Actinobacteriota, and Proteobacteria (Fig. 6A). These five taxa have been reported to be predominant in the canine gut microbiota^48,81^, suggesting that the individuals in this study have a general canine gut microbiota composition. In humans, an increase in Enterobacteriaceae and a decrease in *Bifidobacterium* and *Faecalibacterium* have been reported with aging^41,42^. However, a comparison of the Adult and Senior groups of poodles in this study found no commonality with these reports. No significant differences in beta diversity were detected between the two age groups. We believe that this group of dogs was characterized by a uniform living environment and a uniform diet, with relatively few confounding factors influencing the gut microbiota. Nevertheless, the lack of a significant correlation suggests that while we do not rule out the possibility that some endemic bacterial species may correlate with aging in certain regions or under certain conditions, it is difficult to use the canine gut microbiota as a sensitive marker of chronological aging.

In summary, we found that elevated plasma total protein is a relatively sensitive indicator of chronological aging in healthy small-sized dogs. We also found that NAD⁺ levels and microbiome composition, which have been reported as aging markers in other species, have very low sensitivity as markers of chronological aging, regardless of dog size. Limitations of this study include its single-center design and the limited breeds included. To generalize the aging-related indices to all small dogs, studies should be conducted at multiple institutions and include several small dog breeds other than toy poodles. Future work should measure both ALB and GLO individually and include follow-up periods longer than one year, with subjects stratified into several age categories. Additionally, the relationship between the aging indices identified in this study and the decline in body function with aging needs to be examined in a longitudinal study over a longer time span. Such further efforts will lead to the development of methods for better understanding and preventing aging in dogs.

## Supplementary Information

Supplementary Information.

## Data Availability

16S rDNA sequencing data is available under accession number DRA019236 (under project number PRJDB18730) from the DDBJ DRA database (https://www.ddbj.nig.ac.jp/dra/index-e.html). Other data that support the findings of this study are available from the corresponding authors, T.K.I and S.O., upon reasonable request.
