# Supplementary material for: Plasma protein increase as a chronological aging factor in healthy toy poodles
Source: Sci Rep. 2025 Nov 26;15:42087. doi: 10.1038/s41598-025-26154-2 (PMC12657999; doi:10.1038/s41598-025-26154-2)
Supplement: Supplementary file 1 — Supplementary Information. [file 41598_2025_26154_MOESM1_ESM.pdf]

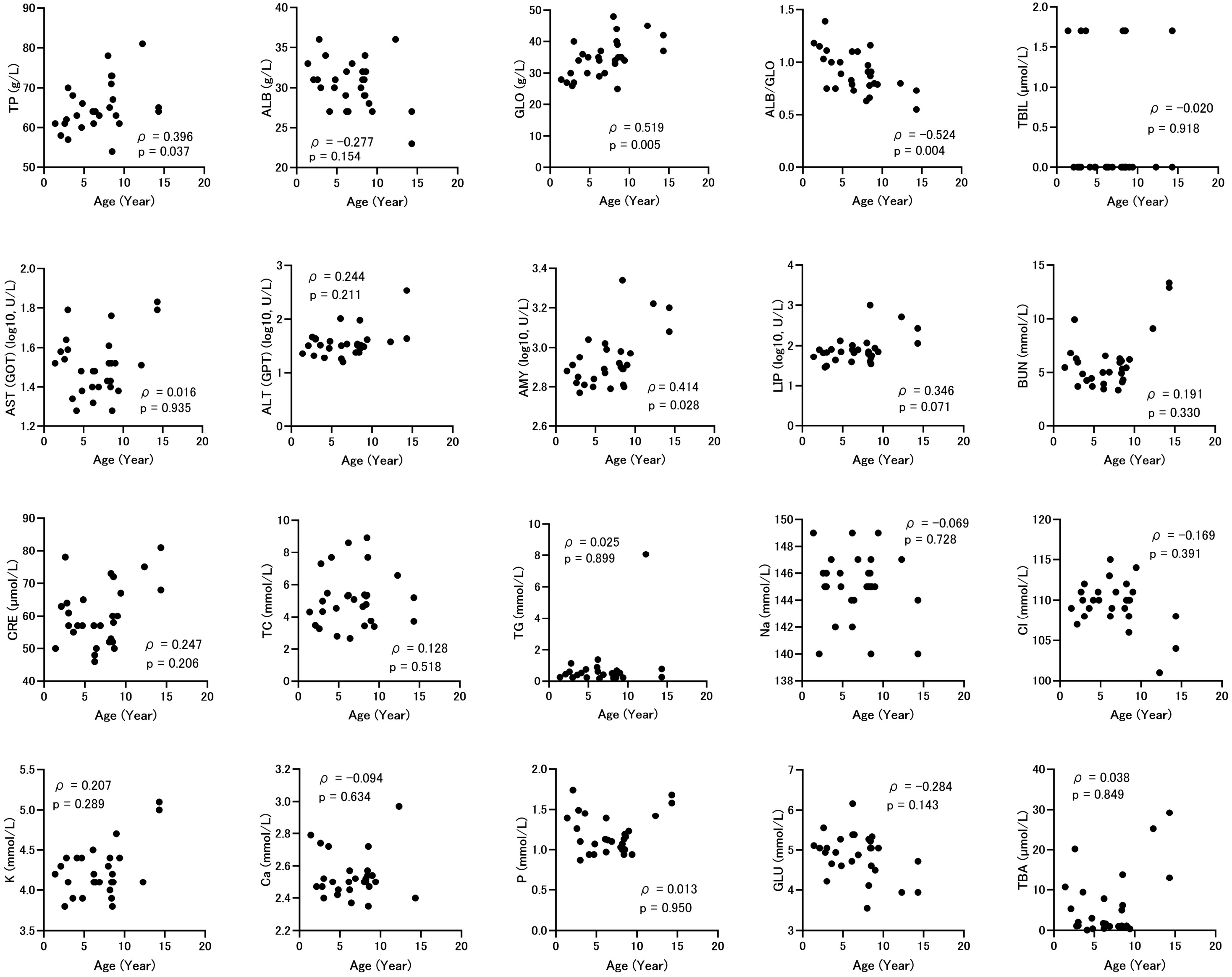

Fig. S1: Relationship between age and blood biochemical indicators in toy poodles at 6 m.
ρ represents the Spearman rank correlation coefficient between age and each indicator.

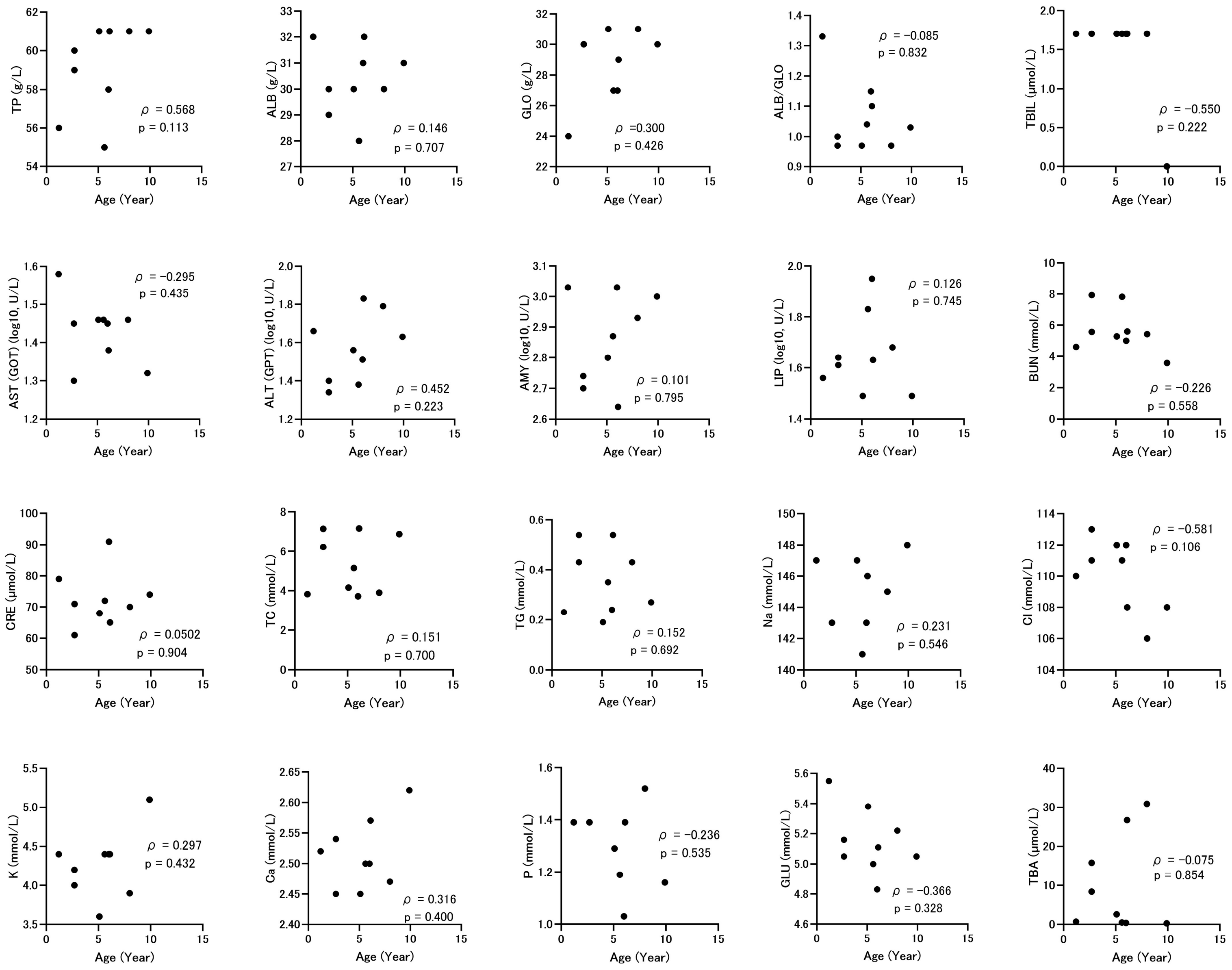

Fig. S2: Relationship between age and blood biochemical indicators in retrievers at 6 m.
ρ represents the Spearman rank correlation coefficient between age and each indicator.

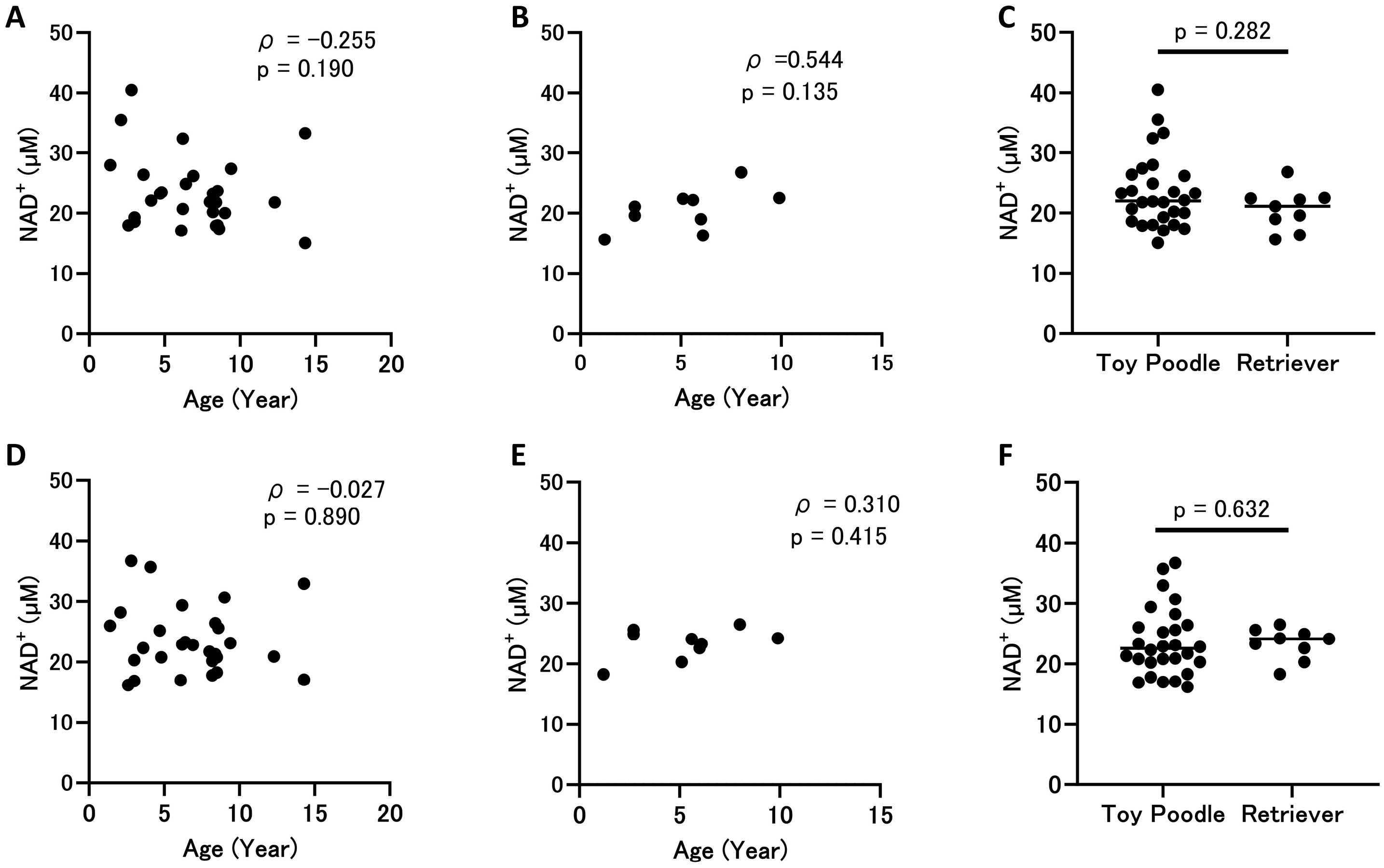

Fig. S3: Relationship between age and whole blood NAD⁺ concentrations.

A, Correlation between age and NAD^+ concentration in the whole blood of toy poodles at 0m. B, Correlation between age and NAD^+ concentration in the whole blood of retrievers at 0m. ρ represents the Spearman rank correlation coefficient between age and NAD^+ concentration. C, Whole blood NAD^+ concentrations in toy poodles and retrievers at 0m. D, Correlation between age and NAD^+ concentration in the whole blood of toy poodles at 6m. E, Correlation between age and NAD^+ concentration in the whole blood of retrievers at 6m. ρ represents the Spearman rank correlation coefficient between age and NAD^+ concentration. F, Whole blood NAD^+ concentrations in toy poodles and retrievers at 6m.

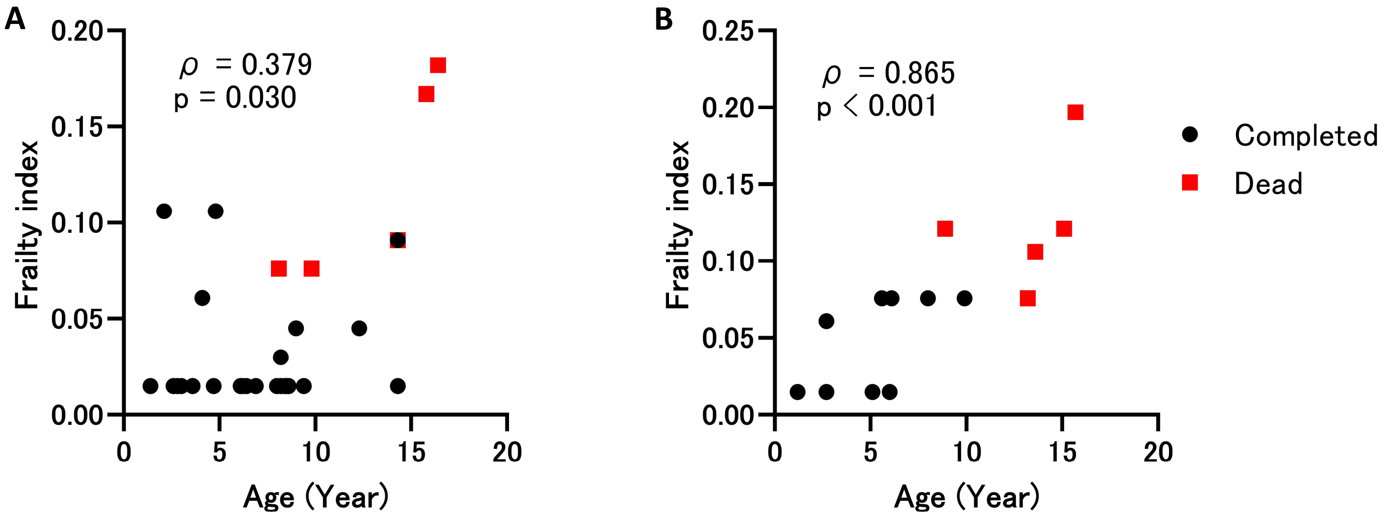

Fig. S4: Relationship between age and frailty index of dogs including individuals deceased during the study.
A, Correlation between age and frailty index at 0 m in toy poodles. B, Correlation between age and frailty index at 0 m in retrievers. Black circles represent individuals who completed the study, and red squares represent individuals who were excluded due to death during the study. Five individuals of each of toy poodles and retrievers died during the study after evaluation for frailty index at 0 m. ρ represents Spearman's rank correlation coefficient for frailty index.

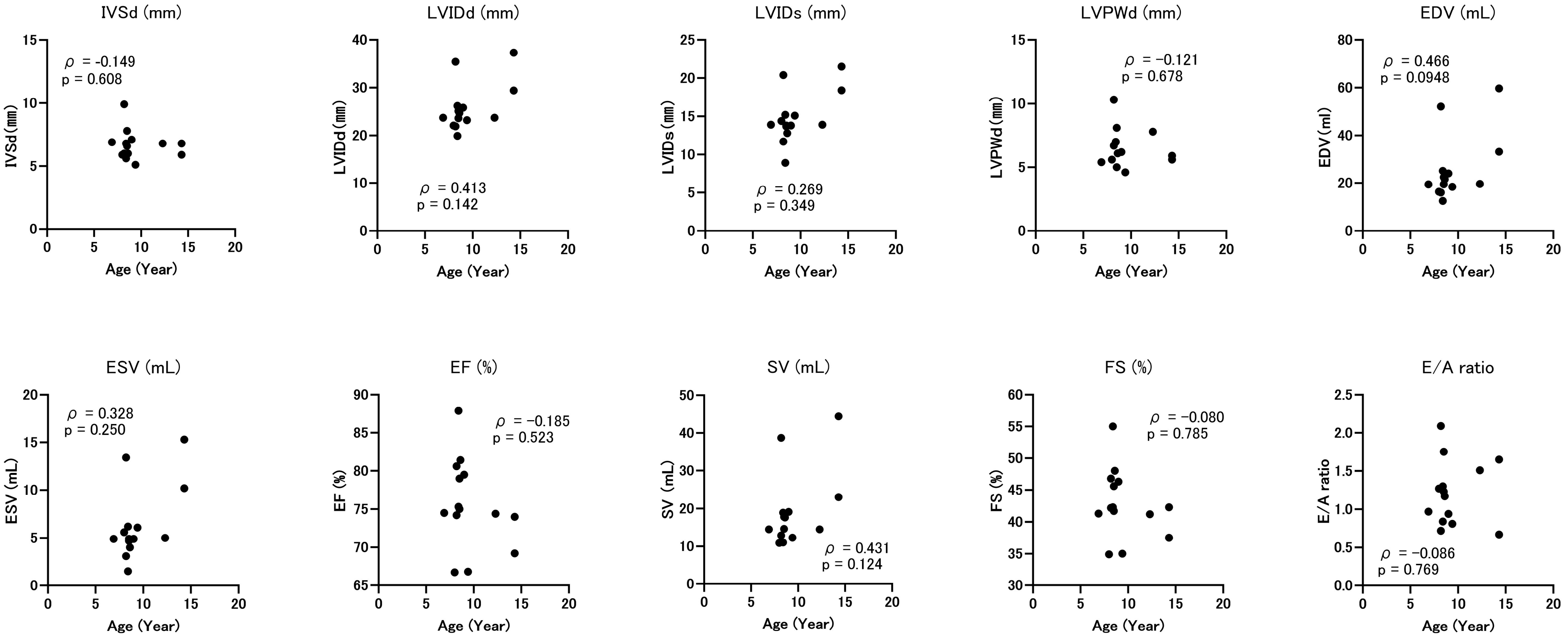

Fig. S5: Relationship between age and echocardiogram indices in toy poodles at 0 m.
ρ represents the Spearman rank correlation coefficient between age and each indicator.

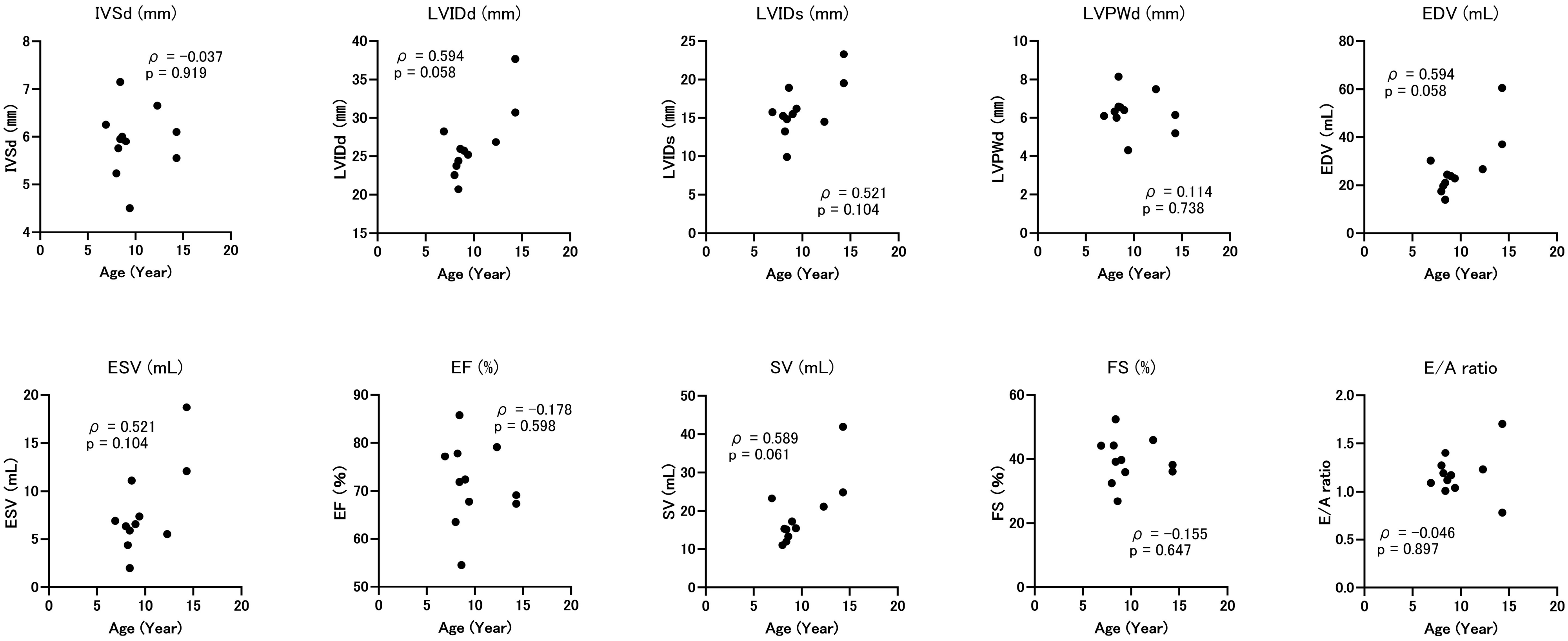

Fig. S6: Relationship between age and the echocardiogram indices in toy poodles at 6 m.
ρ represents the Spearman rank correlation coefficient between age and each indicator.

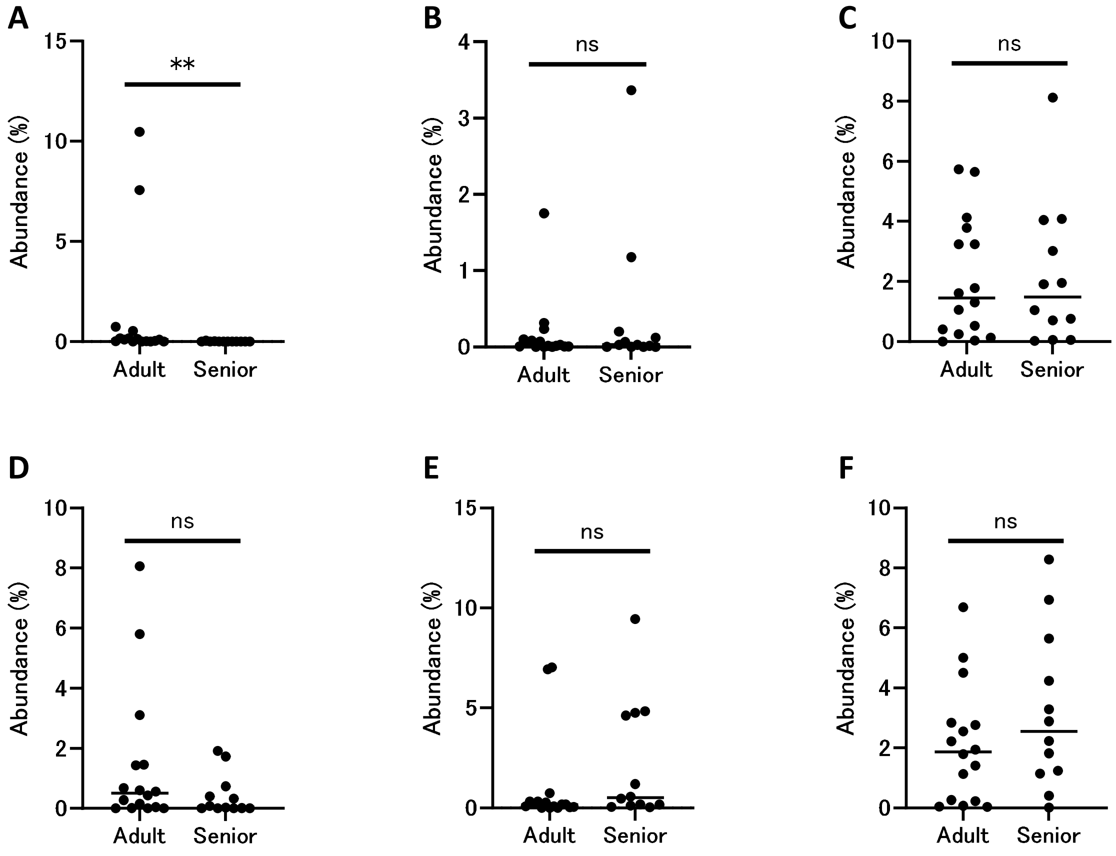

Fig. S7: Relative abundance of each bacterial taxa in fecal microbiota in toy poodles.

A, Bacterial abundance of Enterobacteriaceae at 0 m. B, Bacterial abundance of *Bifidobacterium* at 0 m. C, Bacterial abundance of *Faecalibacterium* at 0 m. D, Bacterial abundance of Enterobacteriaceae at 6 m. E, Bacterial abundance of *Bifidobacterium* at 6 m. F, Bacterial abundance of *Faecalibacterium* at 6 m. The p-values are the results of Mann-Whitney tests to evaluate significant differences between age groups. ** $p < 0.01$. ns: $p > 0.05$.

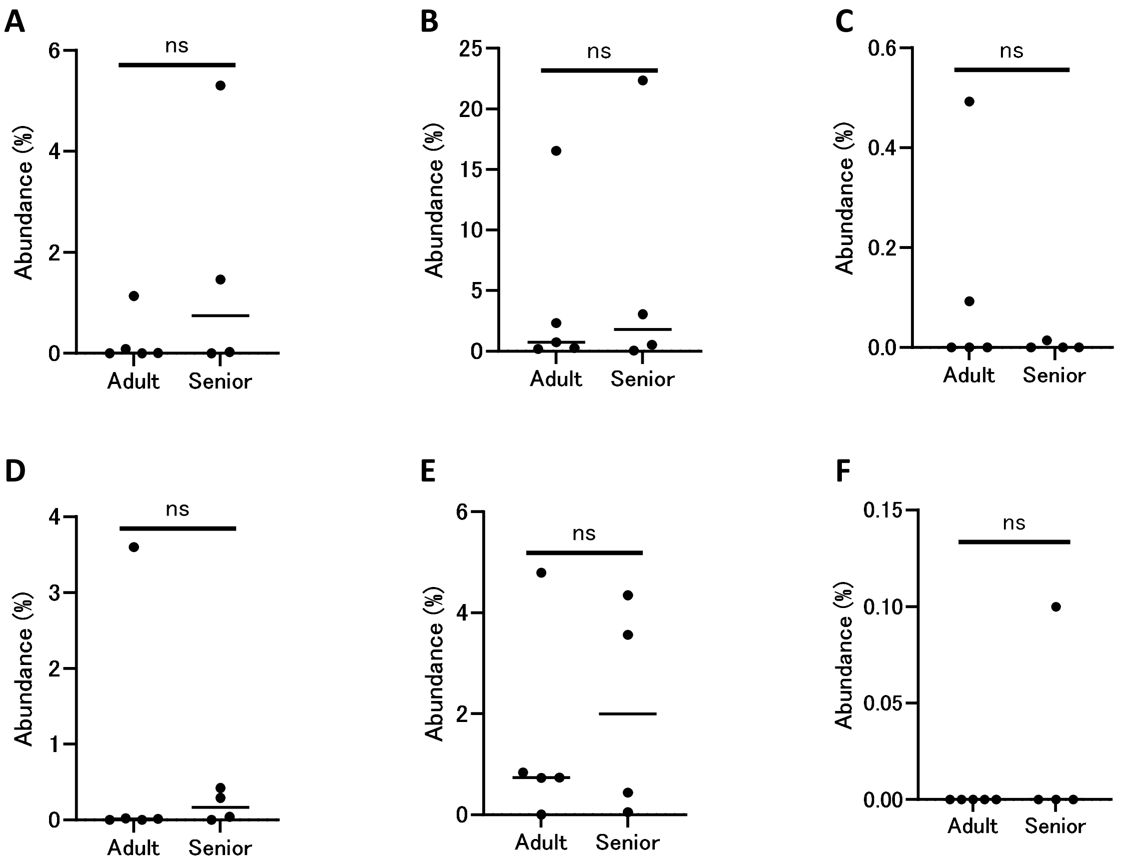

Fig. S8: Relative abundance of each bacterial taxa in fecal microbiota in retrievers.
A, Bacterial abundance of Enterobacteriaceae at 0 m. B, Bacterial abundance of *Bifidobacterium* at 0 m. C, Bacterial abundance of *Faecalibacterium* at 0 m. D, Bacterial abundance of Enterobacteriaceae at 6 m. E, Bacterial abundance of *Bifidobacterium* at 6 m. F, Bacterial abundance of *Faecalibacterium* at 6 m. The Mann-Whitney test was used to test significant differences between the Adult and Senior groups at each time point. ns: $p > 0.05$.
